# Virtual Triggers Real Reactions – Using VR To Assess Youth Violence

**DOI:** 10.1007/s10802-025-01350-w

**Published:** 2025-07-26

**Authors:** Jet Westerveld, Jessica J. Asscher, Hanneke E. Creemers

**Affiliations:** 1 University of Amsterdam. Forensic Child and Youth Care Sciences, Nieuwe Achtergracht 127, Amsterdam, 1018 WS The Netherlands; 2 Utrecht University, Child and Adolescent Studies, Heidelberglaan 1, Utrecht, 3584 CS The Netherlands

**Keywords:** Virtual reality, Juvenile justice, Assessment, Reactive aggression, Self-control and Hostile intent attribution

## Abstract

To improve assessment in forensic youth care, a virtual reality (VR) task was developed to assess behavior without the limitations associated with traditional self-report instruments. The aim of the current study was to examine the potential of this task to assess aggression and its origins, with a focus on hostile intent attribution and low self-control, and its predictive validity in explaining violent infractions during the stay in a juvenile detention facility. Participants were juveniles (*N* = 84; aged 15–23) residing at two all-boys Juvenile Detention Centers in the Netherlands. Responses to four social VR scenarios were observed and, to assess the role of hostile intent attribution (HIA) and low self-control in aggressive responses in these scenarios, participants answered scenario-specific questions about their emotions, thoughts, and motives immediately after each scenario. In addition, self-report questionnaires were used to assess aggression, HIA and self-control. Two months after participation, violent institutional infractions were retrieved from casefiles. Results showed that particularly the more provocative and emotionally engaging scenarios have the potential to elicit aggressive responses. Overall, VR responses and self-report questionnaires showed little convergence, which could not be explained by social desirability nor variation in VR engagement and immersion. Violent institutional infractions were predicted by reactive aggression and low self-control in one of the four scenarios. Concluding, despite little convergence between VR and self-report questionnaires, VR assessment provides potential important information about future violence, which makes it worthwhile to further experiment with and study VR assessment in forensic youth care.

## Introduction

Effectively treating adolescents in forensic care demands a deep understanding of *who* the adolescent is, *what* drives their behavior, and *how* they are most likely to benefit from support. These core ideas are captured in the well-established Risk-Need-Responsivity (RNR) model (Andrews & Bonta, 2010b), which has become a cornerstone for assessment and intervention in offender populations. According to this model, effective treatment must align with three key principles: the risk principle stresses that the intensity of treatment should match the individual’s likelihood of reoffending; the need principle calls for targeting dynamic, changeable risk factors– criminogenic needs– such as aggression; and the responsivity principle emphasizes the importance of delivering interventions in ways that actually reach the adolescent, taking into account their cognitive abilities, emotional state, motivation, and preferred style of learning. Especially in forensic youth care, where many struggle with mistrust and limited insight, tailoring the assessment to the individual is considered essential to increase the likelihood of successful outcomes and decrease the risk of reoffending (Andrews & Bonta, 2010a).

## Assessment with self-report in Forensic Youth Care

To assess factors in the aforementioned domains, numerous screening and risk assessment instruments have been developed to comprehensively assess risk and protective factors (e.g., Barnes-Lee, 2020; Barnoski, 2004; van der Put et al., 2014) or to assess specific risk factors such as mental health problems or aggression (e.g., Achenbach & Rescorla, 2001; Grisso et al., 2001; Raine et al., 2006). Most instruments collect information through questionnaires and interviews with adolescents and thus rely on self-report, whether or not supplemented by third-party and file information. Yet, such instruments often suffer from a number of limitations, which may contribute to their moderate ability to predict future aggressive and delinquent behavior (Kleeven et al., 2022; Koh et al., 2020) and reduce their potential to adequately inform treatment.

Reliable self-report by juveniles in the forensic field is jeopardized by, among others, absence of motivation for completing questionnaires (Kip et al., 2019), youth experiencing difficulties in comprehending and answering to questions due to verbal or intellectual limitations (Hayes et al., 2007), and by youth tending to underreport problems due to concerns about punishment (Kelsey et al., 2015) or poor insight. The latter particularly pertains to aggressive behavior, that has been found to be systematically underperceived in aggressive boys (Lochman & Dodge, 1998). Furthermore, substantial doubt can be raised regarding the capacity of questionnaires or structured interviews to sufficiently capture real-world dynamics and assess actual behaviors, rather than how informants report to feel, think or behave in the past or in a hypothetical situation. As argued by Baumeister et al. (2007), discrepancies between what people say they do and what they actually do indicate that self-reports are not necessarily accurate. This may be particularly relevant for factors that usually occur under conditions of emotional arousal, including aggression and its precursors low self-control and hostile intent attribution; factors commonly targeted in forensic screening. These factors are more likely to be experienced and reported in emotionally engaging situations than generally reported on questions asking about past or hypothetical situations (de Castro et al., 2003; Steinberg, 2004; Verhoef et al., 2021). These limitations may reduce the predictive value of current assessment tools, which is problematic given the RNR-model’s emphasis on accurately identifying dynamic risk factors for treatment aimed at reducing recidivism.

### The Potential of Virtual Reality To Improve Assessment in Forensic Youth Care

To address the limitations of instruments that rely on self-report, we developed an interactive virtual reality (VR) task to investigate the potential of VR to improve assessment in forensic youth care. Interactive VR may be more appealing to youth than completing questionnaires or being interviewed, and therefore allow for involving the youth in forensic assessment without the limitations associated with traditional self-report instruments. In interactive VR, youth can feel immersed, in terms of feeling present in a different reality (Bombari et al., 2015), in a controlled yet emotionally engaging environment in which they interact with and aggress against virtual others, which enhances ecological validity. The standardized task we developed aims to measure the tendency to reactive aggression, defined as the tendency to provide an impulsive aggressive response to perceived threat or provocation (Dodge, 1991), and its background. The design of this VR task was based on the Social Information Processing (SIP) model of aggression (Crick & Dodge, 1994) and Self-Control Theory (Gottfredson & Hirschi, 1990). The SIP model outlines six cognitive steps preceding behavior in social situations: encoding social cues, interpreting those cues, setting interaction goals, generating possible responses, evaluating them, and enacting a chosen response. According to this model, reactive aggression stems from biases in early stages—particularly in interpreting social cues (step 2) and setting aggressive goals (step 3). Youth who habitually interpret ambiguous actions of others as hostile (i.e., hostile intent attribution; HIA) are more likely to experience threat or anger, and subsequently react with impulsive, defensive aggression (Crick & Dodge, 1996). Meta-analyses and longitudinal studies support the link between HIA and aggression (e.g., de Castro et al., 2002; Martinelli et al., 2018; Lansford et al., 2006). Gottfredson and Hirschi’s Self-Control Theory proposes that individuals with low self-control are less capable of restraining themselves from impulses and immediate gratifications, despite potential negative consequences, making self-control the main driver of criminal behavior. Empirical research confirms the link between self-control and behavior (e.g., de Ridder et al., 2012), and specifically between low self-control and aggression when being provoked (DeWall et al., 2007).

Developing the VR task, we designed four social scenarios that may elicit reactive aggression, to measure the tendency to reactive aggression and its origins, i.e., HIA and/or low self-control. In the task, youth are exposed to the different scenarios, their responses to the social triggers are observed and, after each scenario, questions are asked to determine the background of their response. As these questions are asked immediately following each scenario, they call for direct evaluation of discrete behavioral responses, reducing the likelihood of generalized answers or deficient recall. The different scenarios were designed to assess responses in different contexts. In short, the participant is approached by an avatar (scenario #2), the participant is excluded and bullied by two avatars (scenario #3), the participant is provoked and sent away by an avatar (scenario #4) and an authority figure requests the participant to leave (scenario #5). By systematically varying social triggers– ranging from subtle ambiguity to overt hostility– the VR scenarios allow for exploration of how different situational features evoke emotional engagement, hostile intent attribution, challenged self-control and aggressive responses. Scenarios involving clear provocation (e.g., direct verbal or physical threat) can be expected to evoke more immediate aggressive responses (DeWall et al., 2007; Piquero & Rocque, 2020). Other scenario characteristics, such as the presence of authority figures or direct peer provocation, were included to reflect contextual differences shown to influence aggression (e.g., Reijntjes et al., 2011; Gálvez-Nieto et al., 2022). More specifically, because aggressive responses are often more strongly triggered in peer contexts– where social comparison, reputation, and perceived disrespect play a key role (Han et al., 2024; Faris & Ennett, 2012; Laninga-Wijnen et al., 2020) – aggression may be most pronounced in peer conflict scenarios. In contrast, the presence of an authority figure (e.g., teacher or staff member) can be expected to inhibit aggressive behavior compared to peer-only situations, due to increased salience of social norms and anticipated consequences (Gálvez-Nieto et al., 2022; Smeets et al., 2015).

VR technology is already tested for psychological assessment in children and adults (e, g., Emmelkamp & Meyerbröker, 2021; Fromberger et al., 2018; Verhoef et al., 2021). These studies have demonstrated that VR can improve the accuracy and ecological validity of assessments. For example, Emmelkamp and Meyerbröker (2021) found that VR was effective for the assessment of anxiety and post-traumatic stress disorder by immersing participants in emotionally charged environments. Furthermore, Fromberger et al. (2018) showed that VR enhances assessment by creating realistic scenarios that engage participants emotionally, offering a more reliable measure of behaviors compared to traditional methods. However, while the use of VR has been successful in these contexts, its application in forensic youth care remains relatively novel.

## Current Study

To better understand the potential of our VR task for assessment in incarcerated juveniles, the current study aimed to determine the added value of VR assessments in terms of emotional engagement and whether VR assessments allow for assessment of criminogenic factors over and beyond traditional self-report questionnaires. If indeed VR assessment provides additional information, treatment may be better informed of circumstances in which aggressive behavior occurs and what situations may trigger this. To do so, the current study assessed (1) the level of immersion in VR and emotional engagement in the different scenarios and how this relates to behavior in VR; and (2) whether the varying content and levels of provocation in the different social scenarios elicited different levels of emotional engagement, reactive aggression, hostile intent attribution and low self-control. Furthermore, to better understand how measures from VR assessment relate to self-report questionnaire data and future aggression, we first examined (3) associations between measures of reactive aggression, hostile intent attribution, and self-control generated in VR and by self-report questionnaires, and whether these associations are dependent upon socially desirable responding and emotional engagement in VR; and subsequently (4) the predictive validity of measures from the VR scenarios, in terms of explaining violent institutional infractions in the two months after participating in the VR task, and whether VR measures explain additional variance above and beyond self-report questionnaire measures.

## Method

### Participants

**Fig. 1:**
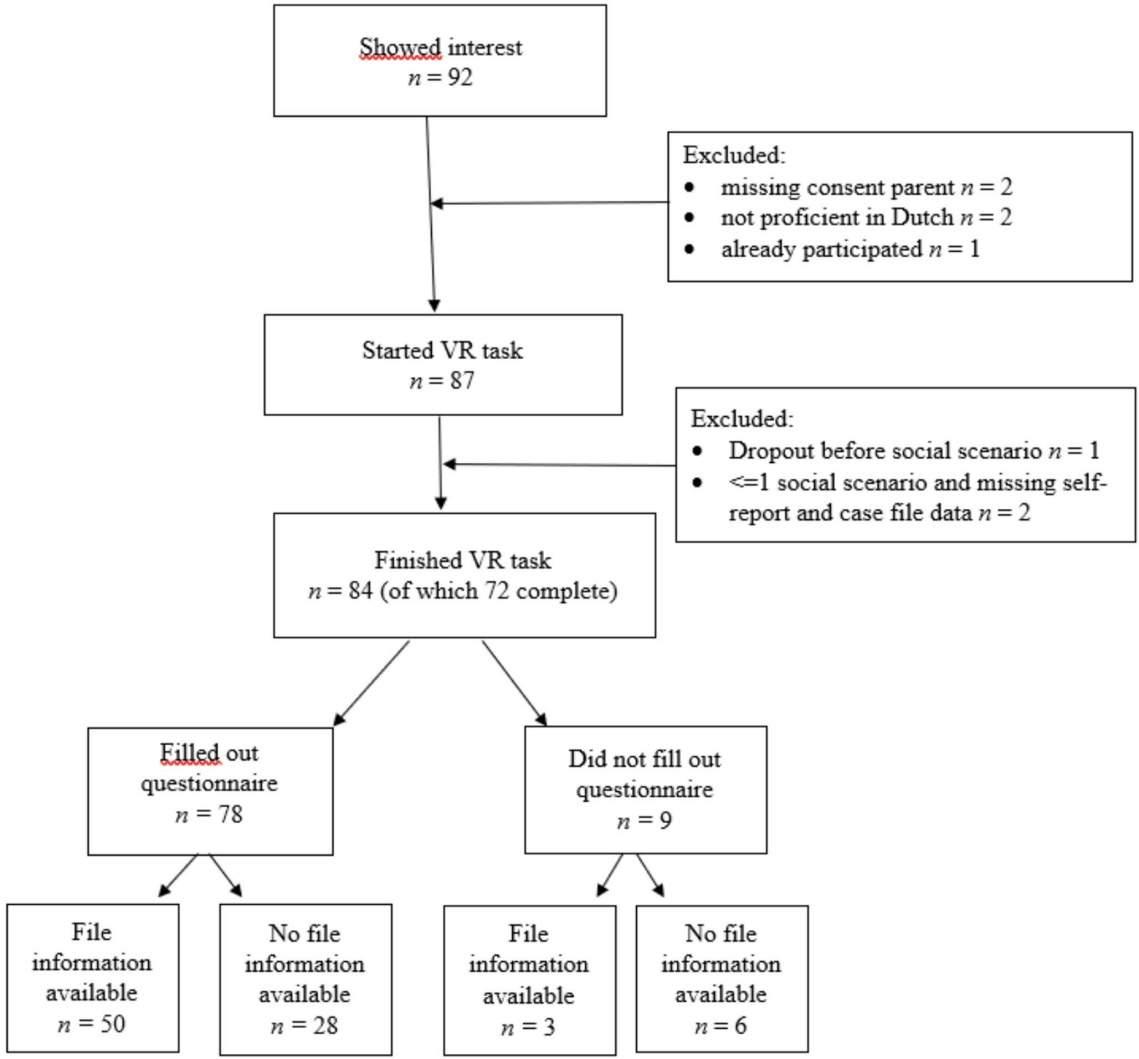
Flowchart recruitment and data collection

Between April and December 2022, recruitment and data-collection for this study took place at two all-boys Juvenile Detention Centers (JDC) in the Netherlands. Once a week, trained research assistants recruited juveniles from short-term stay groups, in which juveniles await their trial after being arrested and referred to a JDC. To be eligible for participation, participants had to be proficient in the Dutch language and have no psychotic or dissociative disruptions. Ninety-two juveniles showed interest to participate. Of these juveniles, four were ineligible because they did not understand nor speak the Dutch language proficiently (*N* = 2), or because they were under the age of 16 and informed consent from a caregiver was missing (*N* = 2). In addition, one juvenile was prevented from participating in the assessment because he had participated in the assessment at a third institution. Consequently, 87 juveniles started the VR task. Three of them dropped out early and were excluded from the analyses because of missing data on all of the social scenarios (*n* = 1) or missing self-report and case file data (*n* = 2). Of the 84 remaining juveniles, 72 (86%) completed all four social scenarios of the VR task and 78 (93%) filled out the self-report questionnaire. Although 95% consented with retrieving information from case files, case file information was only available for 52 juveniles (62%). A flowchart of recruitment and data collection is presented in Fig. 1.

The 84 remaining juveniles were aged 15 to 23 years (M = 17.8, SD = 1.56). At the time of the assessment, 29 participants (34.5%) were enrolled in secondary vocational education (MBO), 16 (19%) in pre-vocational education (VMBO), 4 (4.8%) in practical education (Praktijkonderwijs), and 1 (1.2%) in senior general secondary or pre-university education (HAVO/VWO). Additionally, 14 participants (15.7%) were employed instead of enrolled in education, and 7 (8.3%) were neither in education nor employed. For 13 participants, information regarding education or employment was missing.

## Procedure

Prior to start, this study was approved by the Ethics Review Board of the Faculty of Social and Behavioral Sciences at the University of Amsterdam (2021-CDE-14121) and by the Board of the JDC. On test days, two trained research assistants visited the short-term stay groups and approached juveniles to inform them about the project. They showed a flyer and gave a short explanation of the VR task and the overall project. If a juvenile showed interest and was eligible for inclusion an appointment was made, usually for participation that same day.

The individual assessment took place in a private (treatment) room in the JDC. First, written informed consent was obtained, for youth under the age of 16 also from a caregiver. Then, for participants who also gave permission for cardiac measurements, HR equipment was connected and cardiac functioning was assessed before and throughout the VR task. Because a large proportion of the cardiac data was missing (ranging from 38 to 53% per scenario, cardiac functioning was not further considered for this study. Subsequently, the participant put on a VR-headset (Oculus Rift S, using a single fast-switch LCD panel with a resolution of 2560 × 1440 and an 80 Hz refresh rate with 88 degrees field of view and 6DOF tracking), an audio-headset (participants heard the audio through the headphones in stereo), and held a controller in each hand that enabled them to use their hands. In standing position, the participant ran through the VR task which lasted approximately 25 min. After finishing the task, the participant put down the VR system and was debriefed.

Subsequently, the participant completed a self-report questionnaire, which took approximately 10–15 min. If necessary or asked for, one of the research assistants read the questions out loud while the participant filled out the answers. To conclude, the juvenile was thanked for participation and brought back to the living group of the JDC. Afterwards, the participant received a small monetary stipend (i.e., €5.00 on JDC account) for participation.

### The VR Task

In 2020 and 2021, the VR task was created by the authors and designed by CleVR. It is based on a VR task by Verhoef et al. (2021) to assess aggression in children. An initial version of the task was piloted in 2020 among 29 incarcerated boys in a Juvenile Detention Center (JDC; Krüter et al., 2023). Based on the findings and user experiences in this pilot, the task was further developed and presented to nine boys in forensic youth care. In this phase, both the software and the dialogues were refined to better reflect the lived experience and social perceptions of the target group. These efforts led to the version of the VR task used in the current study.

The interactive task consists of five standardized scenarios to which participants are exposed, in which their behavioural responses are observed and their thoughts about the background of the responses are assessed. All scenarios, one neutral scenario and four social scenarios, took place on a virtual schoolyard after school hours. In the first, neutral scenario (S#1) the participant familiarizes himself in the VR environment and is instructed to play a game on a (virtual) tablet. This game is added to the task to mimic waiting in real life situations, where many people tend to take their smartphone to pass waiting time.

The subsequent social scenarios, that last 60–180 seconds, contain a social trigger that might elicit aggression. The scenarios start with instructions to keep playing the game while waiting for others to arrive. These “others“ are avatars controlled in real time by research assistants, who follow a detailed script and use voice modulators to give each avatar a distinct voice. The participants are not informed that the avatars are operated by the research assistants and are instructed to behave as they would do in real life, and to interact with others as they usually do. In the second scenario (S#2), the participant encounters three other juveniles (i.e., avatars). Two of them sit down on a bench and, after a short time, the third one approaches the participant and coughs in the direction of the participant, while standing close to him. Subsequently, this avatar initiates some small talk, in a prosocial manner. In the third scenario (S#3), the two avatars on the bench exclude and bully the participant. While the participant is playing the aforementioned game, these two avatars start talking to each other about a movie they are watching on their tablets. The “prosocial avatar”, still standing next to the participant, asks if they would like to show the movie to him and the participant as well. The avatar on the bench declines this request, saying– in a provocative manner– that the movie is not “for little boys”. In the fourth scenario (S#4), one of the avatars on the bench provokes the participant. While the participant is again playing the game, this avatar gets up from the bench and comes close to the participant. The avatar challenges the participant by saying that the participant is hanging out in “their little square” and that he has to leave. In the fifth and final scenario (S#5), an authority figure, the principal of the school, appears and kindly asks the participant and the “prosocial avatar” to leave the schoolyard.

During the social scenarios, the research assistant observes the participant’s response to the social trigger and chooses from a set of reply options as mentioned in the script. These replies reflect one of three possible behavioral responses of the participant: provoked/aggressive, neutral/passive, or calm/prosocial. Depending on the participants’ response, the assistant selects the appropriate avatar response. Each social scenario ends with questions to the juvenile about emotions, thoughts and motives that played a role in the juvenile’s reaction (e.g., ‘*How difficult was it to remain calm when the boy provoked you?*’). These questions are answered either by selecting a number on a 10-point scale projected in VR, or by providing an open answer.

After the final scenario, the assessment is concluded with a short conversation including some small talk to make sure that the juvenile is ‘back in the real world’, an evaluation of the assessment to determine the extent to which the juvenile responded as he would do in real life, and debriefing the juvenile by clarifying that the avatars were controlled by the research assistant.

## Measures

### VR Measures

During each of the social scenarios of the VR task, the following measures were collected:

- *Reactive Aggression* was measured by observing participants’ behavioral responses to the social triggers. A trained research assistant made detailed descriptions of participants’ behavioral responses. Subsequently, behavior was independently coded by three research assistants into non-aggressive behavior (e.g., prosocial behavior, avoidance) and reactive aggressive behavior (e.g., coercion, verbal aggression or physical aggression). Agreement for the exclusion and provocation scenario between coders was almost perfect, kappa was 0.85 and 0.98 respectively (Cohen, 1960).
- *Hostile Intent Attribution* was measured with a single item following each social scenario: “The other boy/principle did [behavior of other boy/principle]. To what extent did he do this to bother you, on a scale from 1 meaning *not at all*, to 10, meaning *very*?”. The higher the score, the more hostile the behavior of the avatar is perceived by the participant.
- *Self-control* was measured with a single item following each social scenario: “On a scale from 1 meaning *not at all*, to 10, meaning *very*, how difficult was it for you to remain calm when the other boy/principle did [behavior of other boy/principle]?”. Scores were reverse coded after which higher scores reflected higher self-control.
- *Emotional Engagement* was operationalized as the level of anger experienced by the participant, assessed with a single question after each social scenario: “The other boy/principle did [behavior of other boy/principle]. How angry did this make you feel, on a scale from 1, meaning *not at all*, to 10, meaning *very*?”. The higher the score, the higher the emotional engagement of the participant.
- *Immersion* was assessed with a single question immediately after the VR task: “Did it feel real? Did you feel like you were truly experiencing the events?”. Participants responded on a rating scale from 1 (not at all) to 5 (very), with higher scores reflecting a higher level of immersion.

### Self-report Questionnaire Measures

*Reactive Aggression* was measured with the “reactive aggression” subscale from the Dutch version of the Reactive and Proactive questionnaire (Cima et al., 2013). This scale consists of 11 items, for example ‘*how often have you yelled at others when they were irritating*?, to be answered on a 3-point Likert scale (1 = *never*, 2 = *sometimes*, 3 = *often*). Reactive aggression was computed by averaging the 11 items, with a higher score reflecting higher levels of reactive aggression (α =.86).

*Hostile Intent Attribution* was measured with the subscale “distrust” of the Abbreviated List of Irrational Thoughts (VLIG; Hoogsteder et al., 2014). This subscale consists of 4 items, for example *‘no one can be trusted’*, to be answered on a 6-point Likert scale (ranging from 1 = *totally disagree* to 6 = *totally agree*). A score was computed by averaging the 4 items, with a higher score reflecting more hostile intent attribution (α = 0.67).

*Self-control* was measured with the Dutch translation of the Brief Self-Control Scale (BSCS; Tangney et al., 2004). This scale consists of 13 items (Ferrari et al., 2009) measuring the ability to resist short-term rewards or temptations in order to achieve long-term goals, for example ‘*I refuse things that are bad for me’*. Participants were asked to rate the extent to which the item is applicable to them on a 5-point Likert scale (ranging from 1 = *not at all applicable* to 5 = *very applicable*). A score was computed by averaging the 13 items, with a higher score reflecting a higher level of self-control (α = 0.81).

*Propensity for Social Desirability* was assessed with the Marlowe-Crowne Social Desirability Short Form (MCSD-SF; Ballard, 1992). The MCSD-SF is based on the 33-items of the MCSDS (Crowne & Marlow, 1960) and consists of 13 items. The correlation between the MCSD-SF and the MCSDS is strong, *r* =.93 (Reynolds, 1982). A sample item for social desirability is ‘*I never offend other people’*. Participants answered on a 2-point scale (‘*true’* vs. *‘not true*’). Scores were reverse coded and a total score was computed by averaging the 13 items, with higher scores indicating a higher propensity for social desirability (α =.87).

### Case Files

*The Number of Violent institutional infractions* in the two months after participating in the VR task was retrieved from case files by the researcher. These infractions include instances such as verbal aggression or fighting against other youth or professionals, and are logged by professionals at the JDC. For participants who left the JDC before the two month period had passed (*n* = 29 of *n* = 52 juveniles for which information from case files was available), infractions were retrieved for the period in the JDC after participation.

*Time in JDC after Participating in VR Task*,* in Days*, was calculated by subtracting the date of participation from the date of departure, retrieved from case files. For participants who stayed more than two months after participation, this variable was set at 60 days.

### Statistical Analyses

To provide an overview of the data, descriptive analyses were conducted, calculating mean scores and standard deviations of the variables in this study, in addition to correlations of propensity for social desirability with reactive aggression, hostile intent attribution and self-control, measured in VR and by self-report questionnaires.

Before answering the research questions, imputation of missing data was considered. However, because missingness was not random, we opted for a conservative approach and analyzed available data per analysis, with varying sample sizes depending on the completeness of data for the variables involved. Various analyses were conducted. First, to establish the association of immersion in VR and emotional engagement in the four scenarios with behavior in VR (aim 1), correlations were calculated. Here, correlation with age was also calculated, to determine whether age should be taken into account in subsequent analyses.

Second, to determine whether the different social scenarios elicited different levels of emotional engagement, hostile intent attribution and low self-control (aim 2), repeated measures ANOVAs were performed. In subsequent contrasts, scenario #2 (Approached by peer) was used as a reference because this scenario was considered the most neutral (least triggering) social scenario. McNemar’s tests were used for the dichotomous variable reactive aggression.

Third, to determine the association between measures of reactive aggression, hostile intent attribution and self-control in VR and by self-report questionnaires, and whether these associations were moderated by socially desirable responding and emotional engagement in VR (aim 3), linear hierarchical regression analyses were conducted separately for each of the social scenarios, to account for differences in strength of the associations across scenarios. In these analyses, the variables from self-report questionnaires were the dependent variables, and the variables from VR were the independent variables. Continuous independent variables and moderators were centered around their mean. In the first step, only the independent variable was included; in the second step, the moderator and the interaction between the independent variable and the moderator were included. In case of a significant interaction, a median split on the moderator was used to test the association between self-report and VR measures at low and high levels of the moderator.

Fourth, to examine the predictive validity of measures from the VR scenarios (aim 4), in terms of explaining violent institutional infractions above and beyond self-report questionnaire measures, a hierarchical linear regression analysis was conducted separately for each of the social scenarios. In these regressions, the number of infractions was the dependent variable, and reactive aggression, hostile attribution and self-control in VR were the independent variables included in the first step, while taking the time in the JDC since participation in the VR task into account. In the second step, reactive aggression, hostile attribution and self-control from self-report questionnaires were included in the model.

## Results

### Descriptives

Means and standard deviations of the variables in this study are presented in Tables 1 and 2. Correlations were calculated between propensity for social desirability and reactive aggression, hostile attribution and self-control, as measured in VR and by self-report questionnaires. Adolescents with a stronger propensity for social desirability reported on the self-report questionnaires significantly less reactive aggression (*r* = −.49, *p* <.01), less hostile intent attribution (*r* = −.24, *p* <.05) and more self-control (*r* =.50, *p* <.01). A stronger propensity for social desirability correlated in VR with a higher likelihood of reactive aggression in *scenario #2 *(Approached by peer) (*r* =.27, *p* <.05) and more self-control in scenario #3 (Excluded by peers) (*r* =.24, *p* <.05).

**Table 1:** Descriptive statistics VR task, per social scenario

	*N*	S#2 Approached by peer		S#3 Excluded by peers		S#4 Provoked by peer		S#5 Request from authority figure	
		*M*	*SD*	*M*	*SD*	*M*	*SD*	*M*	*SD*
Engagement (1-10)	70	1.92º	1.73	2.37	2.41	3.69º	2.89	1.62	1.85
Reactive aggression (% yes)	72-75	6.1%¹		25%¹		57.3%¹		27%¹	
Hostile intention attribution (1-10)	70	2.60²³	2.52	6.42²	3.45	8.45³	2.46	2.24	2.72
Self-control (1-10)	70	8.37⁴	2.46	8.11	2.67	7.73	2.85	9.5⁴	1.37

º¹²³⁴ = significant difference

**Table 2:** Descriptive statistics immersion, self-report measures and number of infractions

	*M*	*SD*
VR task		
Immersion VR (1-5)	3.39	1.58
Self-report questionnaire		
Reactive aggression (1-3)	1.75	.42
Hostile intention attribution (1-6)	2.41	1.09
Self-control (1-5)	3.47	.69
Social desirability (1-2)	1.59	.28
Case files		
Number of institutional violent infractions (0-6)	.92	1.20

### Immersion, Emotional Engagement and Behavior in VR

On a scale of 1–5, the mean level of reported immersion in the VR task was *M* = 3.39 (*SD* = 1.58). Immersion did not significantly correlate with reactive aggression, hostile attribution and self-control (Tables 3 and 4). On a scale from 1 to 10, mean levels of emotional engagement ranged from *M* = 1.62 (*SD* = 1.85) in scenario #5 (Request from authority figure) to *M* = 3.69 (*SD* = 2.89) in scenario #4 (Provoked by peer) (Table 1). Higher levels of emotional engagement correlated with lower self-control (all scenarios), with more hostile intent attribution (scenarios #3, #4 and #5) and with reactive aggression (scenario #4). Immersion, engagement and behavior in VR did not correlate with age, with one exception. Only in scenario #5 (Request from authority figure), a significant correlation indicated that older boys were less likely to show reactive aggression (*r* = −.29, *p* <.05). Given these correlations, age was not further considered as a covariate.

**Table 3:** Correlations VR task scenario S#2 (Approached by peer; below diagonal) and S#3 (Excluded by peers; above diagonal)

	1.	2.	3.	4.			5.	6.
1. Immersion		0.08	0.19		−0.14	−0.05		0.03
2. Engagement	−0.02		0.35**		−0.54***	0.17		−0.05
3. Hostile intention attribution	−0.03	0.16			−0.26*	0.06		0.03
4. Self-control	−0.11	−0.27*	−0.12			−0.12		−0.03
5. Reactive aggression	−0.12	0.18	−0.07		0.08			−0.14
6. Age	0.03	0.10	−0.02		−0.21	0.01		

*p <.05; ** *p* <.01; ***p <.001

**Table 4:** Correlations VR task scenario S#4 (Provoked by peer; below diagonal) and S#5 (Request from authority figure; above diagonal)

	1.	2.	3.	4.			5.	6.
1. Immersion		−0.10	0.02		0.20	−0.08		0.03
2. Engagement	0.33**		0.25*		−0.51***	0.12		−0.10
3. Hostile intention attribution	0.05	0.26*			−0.56***	0.32**		−0.05
4. Self-control	−0.16	−0.66***	−0.21			−0.23		0.08
5. Reactive aggression	0.19	0.32**	−0.01		−0.27*			−0.29*
6. Age	0.03	−0.06	−0.12		0.01	0.10		

*p <.05; ** *p* <.01; ***p <.001

### Differential Functioning of the Social Scenarios

Repeated measures ANOVAs showed that the social scenarios varied in the level to which they elicited engagement (*F*(3, 207) = 14.2, *p* <.001) and hostile intent attribution (*F*(2.4, 166; degrees of freedom were corrected using Greenhouse-Geisser/Huynh-Feldt because the assumption of sphericity had been violated) = 110.2, *p* <.001) and challenged self-control (*F*(3, 207) = 8.4, *p* <.001). Compared to scenario #2 (Approached by peer), scenario #4 (Provoked by peer) elicited more engagement, scenario #3 (Excluded by peers) and scenario #4 (Provoked by peer) elicited more hostile intent attribution, and scenario #5 (Request from authority figure) resulted in a higher average level of self-control. McNemar tests showed that scenarios #3-#5 elicited more reactive aggression than scenario #2 (Approached by peer), all *p*’s < 0.001 (Table 1).

### VR and Self-Report Measures

For reactive aggression, there were no direct associations between measures from self-report questionnaires and VR, with one exception (Table 5). Reactive aggression in VR scenario #2 (Approached by peer) was associated with lower levels of self-reported reactive aggression, *F* (1,73) = 7.48, *p* <.01, *R²* =0.09. When interpreting this result, it should be taken into account that reactive aggression was relatively unlikely in scenario #2 (Approached by peer). The associations between VR and self-report measures were not moderated by propensity to social desirability or emotional engagement.

**Table 5:** Associations between measures in VR and self-report measures

		Reactive aggression			Hostile intent attribution			Self-control		
	*N*	*B*	*SE*	*Beta*	*B*	*SE*	*Beta*	*B*	*SE*	*Beta*
S#2: Approached by peer	75	− 0.51**	0.19	− 0.31	− 0.14	0.13	− 0.13	0.08	0.09	0.11
S#3: Excluded by peers	67	0.05	0.12	0.05	0.10	0.14	0.09	0.24**	0.08	0.34
S#4: Provoked by peer	69	0.10	0.10	0.12	0.13	0.14	0.11	0.15	0.09	0.21
S#5: Request from authority figure	68	− 0.02	0.12	− 0.02	− 0.03	0.13	0.03	0.06	0.09	0.09

*p <.05; ** *p* <.01

For hostile intent attribution, there were no direct associations between self-report questionnaires and VR (Table 5). In scenario #3 (Excluded by peers), the propensity for social desirability was a significant moderator. Yet, post hoc analyses showed that neither at low nor high levels of social desirability, VR and questionnaire measures correlated significantly. In scenario #4 (Provoked by peers), the level of emotional engagement was a significant moderator. At low levels of emotional engagement, hostile intent attribution in VR was positively associated with reported hostile intent attribution in the questionnaire *F* (1,30) = 4.51, *p* <.05, *R²* =0.13, whereas this association was not significant at high levels of engagement *F* (1,36) = 1.90, *p* >.05.

For self-control, there were no direct associations between measures from self-report questionnaires and VR, with one exception (Table 5). Self-control in VR scenario #3 (Excluded by peers) was positively associated with self-control reported in the questionnaire, *F* (1,64) = 9.94, *p* <.01, *R²* =0.13. Propensity for social desirability did not moderate the association between VR and questionnaire measures of self-control. In scenario #4, the level of emotional engagement was a significant moderator. Yet, post hoc analyses showed that neither at low nor high levels of engagement, VR and questionnaire measures correlated significantly.

### VR and Violent Institutional Infractions

Reactive aggression and low self-control in VR scenario #3 (Excluded by peers) predicted a higher number of violent institutional infractions in the two months after participating in the VR task, *F* (4,38) = 4.90, *p* <.01, *R²* =0.34. Significant results were retained when self-report questionnaire measures were included in the model (Table 6). Hostile intent attribution, reactive aggression and self-control in any of the other scenarios did not predict violent institutional infractions.

**Table 6:** Associations between measures in VR and violent institutional infractions

	S#2 Approached by peers (*N* = 46)			S#3 Excluded by peers (*N* = 43)			S#4 Provoked by peer (*N* = 44)			S#5 Request from authority figure (*N* = 44)		
	*B*	*SE*	*Beta*	*B*	*SE*	*Beta*	*B*	*SE*	*Beta*	*B*	*SE*	*Beta*
Step 1– VR measures												
Reactive aggression	1.14	0.76	0.23	1.00**	0.32	0.41	0.77	0.42	0.30	0.31	0.51	0.11
Hostile intent attribution	0.13	0.21	0.10	− 0.01	0.14	− 0.01	0.05	0.19	0.04	− 0.24	0.24	− 0.20
Self-control	0.02	0.19	0.02	− 0.32*	0.15	− 0.29	− 0.18	0.22	− 0.13	0.01	0.27	0.01
Time spent in JDC after participation	0.02	0.02	0.20	0.02	0.01	0.21	0.03	0.02	0.29	0.02	0.02	0.20
Step 2												
Reactive aggression	1.92*	0.83	0.39	0.91**	0.32	0.38	0.73	0.43	0.29	0.20	0.53	0.07
Hostile intent attribution	0.24	0.21	0.18	0.09	0.14	0.09	0.11	0.21	0.09	− 0.18	0.25	− 0.14
Self-control	0.01	0.19	0.01	− 0.34*	0.17	− 0.31	− 0.18	0.24	− 0.13	0.14	0.30	0.09
Time spent in JDC after participation	0.01	0.02	0.11	0.02	0.01	0.20	0.03	0.02	0.31	0.02	0.02	0.18
Reactive aggression questionnaire	0.29	0.60	0.09	− 0.58	0.45	− 0.22	− 0.63	0.77	− 0.18	− 0.14	0.72	− 0.04
Hostile intent attribution questionnaire	0.37	0.19	0.37	0.28*	0.13	0.35	0.28	0.21	0.28	0.32	0.22	0.31
Self-control questionnaire	0.12	0.35	0.06	0.19	0.25	0.12	0.09	0.38	0.04	0.13	0.40	0.07

*p <.05; ** *p* <.01

## Discussion

### Emotional Engagement

The first aim of this study was to determine, in a group of 84 boys placed in a juvenile detention center, the experienced immersion and emotional engagement and their relation to behavior in VR. The average level of immersion, i.e., whether youth felt present in a new reality, reported by the juveniles, was between “a little” and “neutral”. The level of immersion in the current study seemed slightly less than the one observed in the study conducted by Verhoef et al. (2022), which focused on boys aged 7–13 years and served as the foundation for the task developed in our study. As judgement increases with development, it is plausible that age plays a role in the somewhat lower levels of immersion in the current study. Level of immersion did not correlate with reactive aggression, hostile attribution and low self-control, suggesting that high immersion is no precondition for displaying such behaviors in VR.

Achieving substantial emotional engagement in the scenarios in this task, indicated by the experienced level of anger, was considered crucial to capture behaviors that may occur in real-life situations (Steinberg, 2004). In our VR task, juveniles reported fairly low levels of emotional engagement. Verhoef et al. (2022) found higher levels of emotional engagement in 7–13 year old boys high on disruptive behavior problems when exposed to an interactive VR task to assess aggressive social information processing and responses. The older age of the sample in the current study compared to that in Verhoef et al. may be a potential explanation, because throughout adolescence and young adulthood the experience of anger decreases (Blanchard-Fields & Coats, 2008). Despite the on average fairly low level of emotional engagement in the current study, individual differences in engagement were observed. Correlations of higher levels of emotional engagement with lower levels of self-control, more hostile intent attribution and reactive aggression, which were stronger in the more provocative scenarios, confirmed that these behaviors are more likely to be presented under conditions of emotional arousal.

### Differences Between Scenarios

The second aim was to explore whether the varying content and levels of provocation in the different social scenarios elicited different levels of emotional engagement, reactive aggression, hostile intent attribution and low self-control. Our findings suggest that the scenarios were experienced differently by the participants. As expected (DeWall et al., 2007; Piquero & Rocque, 2020), the scenarios involving clear provocation, i.e., scenarios #3 (Excluded by peers), #4 (Provoked by peer), and #5 (Request from authority figure), evoked more immediate aggressive responses than the more neutral scenario #2 (Approached by peer). The two scenarios involving provoking peers were experienced as most hostile. Both scenarios involve clear norm violations and threats to the participant’s autonomy or social status, which are particularly salient triggers for anger and reactive aggression during adolescence (DeWall et al., 2007). In scenario #3, the participant is openly mocked or excluded by a peer, which may evoke strong feelings of social injustice or humiliation and increase the urge to retaliate. In scenario #4, a peer illegitimately asserts authority by sending the participant away from a public space — a direct and confrontational interaction that may be perceived as both disrespectful and threatening. These types of provocations are known to elicit stronger emotional and behavioral responses, particularly in youth with heightened sensitivity to perceived social threats (Crick & Dodge, 1996; Dodge & Coie, 1987). This aligns with theories on situational triggers of aggression (DeWall et al., 2007); Piquero et al., 2016), which suggest that aggression is more likely when individuals experience social exclusion, disrespect, or a challenge to their social standing. These findings are also in line with prior empirical studies showing that both hostile intent attribution and aggressive responses increase in contexts where individuals feel personally attacked or belittled (Klein Tuente et al., 2019; Verhoef et al., 2019). While scenario #5 also triggered more reactive aggression compared to scenario #2 (Approached by peer), it was not experienced more hostile. Rather, it resulted in the experience of more self-control. The involvement of an authority figure who approaches the participant in a prosocial manner, may have inhibited stronger reactions. In such contexts, youth may be more conscious of consequences or social norms, which might explain why they reported greater self-control in that scenario. Overall, these findings support the theoretical expectation that aggressive responses are more likely when youth feel personally attacked or socially undermined, and underscore the relevance of scenario #3 (Excluded by peers) and #4 (Provoked by peer) for eliciting such responses in VR-based assessments.

#### Convergence of VR and Self-Report Measures

Third, we aimed to understand how VR and self-report measures relate, to gain a comprehensive assessment of reactive aggression and it’s precursors. Overall, there was little convergence between measures generated by VR and by self-report questionnaires. Furthermore, although a propensity to social desirability and emotional engagement were associated with, respectively, more and less favorable self-reports, social desirability nor emotional engagement convincingly explained the absence of convergence between VR and questionnaire measures. A potential explanation is that interactive VR and self-report tap different aspects of aggression and related concepts, which hampers comparability. For instance, in VR, participants are actively exposed to virtual situations where aggressive behavior can be elicited, while self-report requires participants to retrospectively reflect on their aggressive behaviors in the past. These disparate measurement approaches may result in perceptual and reporting differences regarding aggressive behavior, potentially leading to a lack of convergence between the two methods (Steinberg, 2004). Consistent with previous literature highlighting the limitations of self-report questionnaires (Kip et al., 2019), our study’s results align with the notion that questionnaires filled in by a forensic target group are susceptible to social desirability bias. These findings underscore the challenges inherent in relying solely on traditional self-report measures, as they may not fully capture the complexity and authenticity of behavioral responses, particularly in the context of aggression assessment among juvenile forensic populations. Our findings suggest that the interactive VR task has triggered responses, predominantly devoid of socially desirable answers, which may manifest most at higher levels of emotional engagement.

### Predictive Validity of VR Measures

Last, we aimed to determine the predictive validity of measures from the VR scenarios in explaining violent institutional infractions in the two months after participating in the VR task. Juveniles who showed reactive aggression and low self-control in VR scenario #3 (Excluded by peers) had more infractions in the two months following participation in the VR task, and these VR measures surpassed the predictive power of questionnaire measures. As such, scenario #3 (Excluded by peers) seems most apt to differentiate juveniles who are easily offended and respond with aggression from those who do not. The avatar in scenario #3 (Excluded by peers) employs a more indirect, insulting form of aggression compared to the other scenarios. Prior research suggests that individuals who are easily offended or perceive insults and belittlement more intensely tend to respond more aggressively (Reijntjes et al., 2011). Their heightened emotional reactivity and sensitivity to perceived slights can lower the threshold for aggressive reactions when they encounter situations that challenge their self-esteem or social standing. This emotional vulnerability may elicit reactive aggression as a way to protect their self-image or assert dominance in response to perceived threats.

Building on the theoretical framework introduced earlier, the present findings can be further contextualized within the RNR model (Andrews & Bonta, 2010a). The predictive validity demonstrated in scenario #3 reflects the *Risk principle* by identifying juveniles at increased risk for future aggressive behavior. The VR task’s ability to uncover specific situational and emotional triggers aligns with the *Need principle*, as these individualized factors can serve as focal points for targeted interventions. Moreover, the detailed behavioral and emotional data gathered through VR support the Responsivity principle by facilitating the tailoring of treatment approaches to the juveniles’ unique cognitive and emotional profiles, thereby potentially enhancing engagement and effectiveness. Thus, these results reinforce that VR-based assessments may have added value in operationalizing the RNR model within forensic youth care.

## Conclusion

In sum, this study shows that our interactive VR task, and particularly the more provocative and emotionally engaging scenarios, have the potential to elicit aggressive responses in juveniles, supporting the value of VR for assessing reactive aggression. When comparing the VR responses with self-report questionnaires assessing similar constructs, a lack of convergence was demonstrated, suggesting that VR assessment and self-report questionnaires tap different aspects of reactive aggression, hostile intent attribution and self-control. The associations between reactive aggression and self-control in VR and violent institutional infractions two months later demonstrate some predictive validity of the VR task.

### Limitations and Future Directions

This study was the first to examine the potential of a novel application of VR for assessment of reactive aggression and its origins in juveniles in forensic youth care. This innovative approach represents a significant strength of this study, as it offers valuable insights that complement and expand upon the limitations of standard (criticized) assessment methods within forensic youth care, particularly self-report instruments. Another strength of this study is that assessments took place in real-life clinical practice in a hard to reach population showing aggression on regular base. This not only increases external validity, but also provides unique insights in reaction patterns of this relatively understudied population and allows for comparison between self-reported and elicited behavior.

Several limitations should also be taken into account. First, our sample consisted of incarcerated 15–23 year-old boys proficient in the Dutch language and we did not collect data on race, ethnicity, and type of arrests (violent or non-violent). This hampers generalizability of findings and may mask disparities in how aggression is assessed or expressed. Future studies should systematically collect and report this information, especially given the broader concerns about equity and fairness in forensic evaluations. Second, although there is conceptual overlap between the VR and self-report measures of reactive aggression, hostile intent attribution and self-control, particularly the latter two self-report measures did not optimally tap the construct of interest. This may have contributed to not finding significant associations between VR and self-report measures. In addition, although we supplemented self-report questionnaires with casefile information about violent institutional infractions, we did not collect questionnaire or observational data from caregivers, nor data from (violence) risk assessment instruments. Comparison with additional measures that are less subject to bias would have enabled further understanding of the responses in VR and their value for the prediction of future behavior and treatment. As such, we recommend future comparative studies between VR-based assessments and other established methods. These comparisons will contribute to the validation of VR as a tool for assessing aggression and related constructs in forensic youth care settings to improve the diagnostic process. Third, our VR task requires further attention and adjustment in order to achieve more emotional engagement in this target group as well as better capture the constructs of interest. With regard to the latter, emotional engagement was operationalized as self-reported anger in response to a social trigger, measured by a single item. While anger is a relevant affective indicator in aggression research, it captures only one dimension of emotional involvement. Furthermore, self-control was indicated by the single item “how difficult was it for you to remain calm when the other boy/principle did [behavior of other boy/principle]?”, irrespective of the actual behavioral response that followed. Yet, a participant who experiences great difficulty but refrains from aggression may have more self-control than one who experiences no difficulty. Future research should pay close attention to this, as well as systematically test differences between the scenarios and between engagement and behavioral responses in the different scenarios to come to a better understanding. Fourth, although the sample size of this study is quite substantial for innovative, experimental research in this population, it may have limited statistical power to find small associations, particularly for the analyses on violent infractions due to a large proportion of missing casefile information. Taken together, these limitations point to important directions for future research. To better understand and refine the diagnostic utility of VR assessments, future studies should compare VR with both other standardized measures (e.g., observational data, caregiver reports) and physiological or implicit markers of aggression and self-control. Replication with more diverse samples—across gender, cultural backgrounds, and offense types—will also be crucial to test the generalizability and fairness of VR assessments. Finally, refining the VR task by enhancing the realism of scenarios, expanding the emotional measurement framework, and exploring the impact of individual traits such as cognitive functioning or trauma history, could further strengthen its utility in forensic diagnostics.

### Clinical Implications

Especially in the target group of juvenile offenders, it is helpful for treatment and crucial for societal safety to be able to predict future aggression and violence. This study suggests it is worthwhile to further experiment with the use of VR to diagnose aggression and replicate the current findings. Particularly the results of scenario #3, combined with the high levels of social desirability on self-report questionnaires, convincingly show that VR assessments provide potential important information about future aggression, over the prediction made based on self-report questionnaires. This has direct implications for clinical practice, as understanding the situational triggers of aggression—particularly in emotionally provocative contexts—can help therapists identify youths at greater risk of violent behavior. For example, insights from VR assessments can guide individualized treatment planning by informing clinicians about the types of scenarios that elicit strong emotional and behavioral reactions in specific clients. Additionally, VR may serve as a useful tool to assess treatment progress or responsiveness over time, especially for youth who tend to underreport their difficulties. In the context of risk assessment and decision-making, such as sentencing, detention, or conditional release, incorporating VR tasks could support a more dynamic and ecologically valid assessment of behavioral risk, ultimately contributing to both individual rehabilitation and public safety. Experimenting and implementing VR use more broadly may require extra funding and effort of facilities, which can be at odds with budget and personnel limitations. Therefore, we suggest to start with small scale implementation and project based extra funding. Once the added value becomes more evident, more structural resources may become available.

## Data Availability

Data available from last author on reasonable request.

## References

[CR1] Achenbach, T. M., & Rescorla, L. A. (2001). *Manual for the ASEBA school-age forms & profiles*. University of Vermont, Research Center for Children, Youth, & Families.

[CR2] Andrews, D. A., & Bonta, J. (2010a). *The psychology of criminal conduct* (5th ed.). Routledge.

[CR3] Andrews, D. A., & Bonta, J. (2010b). The Risk-Need-Responsivity Model for offender assessment and rehabilitation. In D. P. Farrington & B. C. Welsh (Eds.), *The Oxford handbook of crime prevention* (pp. 267–294). Oxford University Press.

[CR4] Ballard, R. (1992). Short forms of the Marlowe-Crowne social desirability scale. *Psychological Reports*, *71*(3_suppl), 115–1160. 10.2466/pr0.1992.71.3f.1155 10.2466/pr0.1992.71.3f.1155 1480695

[CR5] Barnes-Lee, A. R. (2020). Development of protective factors for reducing juvenile reoffending: A strengths-based approach to risk assessment. *Criminal Justice and Behavior*, *47*(11), 1371–1389. 10.1177/0093854820949601

[CR6] Barnoski, R. P. (2004). *Assessing Risk for Re-offense: Validating the W ashington State Juvenile Court Assessment *(Report No. 04-03-1201). Olympia: Washington State Institute for Public Policy. https://wsipp.wa.gov/ReportFile/869

[CR7] Baumeister, R. F., Vohs, K. D., & Funder, D. C. (2007). Psychology as the science of self-reports and finger movements: Whatever happened to actual behavior? *Perspectives on Psychological Science*, *2*(4), 396–403. 10.1111/j.1745-6916.2007.00051.x 26151975 10.1111/j.1745-6916.2007.00051.x

[CR8] Blanchard-Fields, F., & Coats, A. H. (2008). The experience of anger and sadness in everyday problems impacts age differences in emotion regulation. *Developmental Psychology*, *44*, 1547–1556. 10.1037/a0013915 18999321

[CR9] Bombari, D., Schmid Mast, M., Canadas, E., & Bachmann, M. (2015). Studying social interactions through immersive virtual environment technology: Virtues, pitfalls, and future challenges. *Frontiers in Psychology*, *6*, 869. 10.3389/fpsyg.2015.00869 26157414 10.3389/fpsyg.2015.00869 PMC4478377

[CR10] Cima, M., Raine, A., Meesters, C., & Popma, A. (2013). Validation of the Dutch reactive proactive questionnaire (RPQ): Differential correlates of reactive and proactive aggression from childhood to adulthood. *Aggressive Behavior,**39*(2), 99–113. 10.1002/ab.21458 23386470 10.1002/ab.21458

[CR11] Cohen, J. (1960). A coefficient of agreement for nominal scales. *Educational andPsychological Measurement,**20*(1), 37–46. 10.1177/001316446002000104

[CR12] Crick, N. R., & Dodge, K. A. (1994). A review and reformulation of social-information-processing mechanisms in children’s social adjustment. *Psychological Bulletin*, *115*, 74–101. 10.1037/0033-2909.115.1.74

[CR13] Crick, N. R., & Dodge, K. A. (1996). Social information-processing mechanisms in reactive and proactive aggression. *Child Development*, *67*(3), 993–1002. 10.1111/j.1467-8624.1996.tb01778.x 8706540

[CR14] Crowne, D. P., & Marlowe, D. (1960). A new scale of social desirability independent of psychopathology. *Journal of Consulting Psychology,**20*(4), 349–354. 10.1037/h0047358 10.1037/h0047358 13813058

[CR15] de Castro, B. O., Slot, N. W., Bosch, J. D., Koops, W., & Veerman, J. W. (2003). Negative feelings exacerbate hostile attributions of intent in highly aggressive boys. *Journal of Clinical Child and Adolescent Psychology*, *32*(1), 56–65. 10.1207/S15374424JCCP3201_06 12611030 10.1207/S15374424JCCP3201_06

[CR16] de Castro, B. O., Veerman, J. W., Koops, W., Bosch, J. D., & Monshouwer, H. J. (2002). Hostile attribution of intent and aggressive behavior: A meta-analysis. *Child Development*, *73*(3), 916–934. 10.1111/1467-8624.00447 12038560 10.1111/1467-8624.00447

[CR17] de Ridder, D. T., Lensvelt-Mulders, G., Finkenauer, C., Stok, F. M., & Baumeister, R. F. (2012). Taking stock of self-control: A meta-analysis of how trait self-control relates to A wide range of behaviors. *Pers Soc Psychol Rev*, *16*(1), 76–99. 10.1177/1088868311418749 21878607 10.1177/1088868311418749

[CR18] DeWall, C. N., Baumeister, R. F., Stillman, T. F., & Gailliot, M. T. (2007). Violence restrained: Effects of self-regulation and its depletion on aggression. *Journal of Experimental Social Psychology*, *43*(1), 62–76. 10.1016/j.jesp.2005.12.005

[CR19] Dodge, K. A. (1991). The structure and function of reactive and proactive aggression. In D. J. Pepler & K. H. Rubin (Eds.), *The development and treatment of childhood aggression* (pp. 201–218). Hillsdale, NJ: Lawrence Erlbaum Associates.

[CR20] Dodge, K. A., & Coie, J. D. (1987). Social-information-processing factors in reactive andproactive aggression in children’s peer groups. *Journal of Personality and Social Psychology,**53*(6), 1146–1158. 3694454 10.1037//0022-3514.53.6.1146

[CR21] Emmelkamp, P. M., & Meyerbröker, K. (2021). Virtual reality therapy in mental health. *Annual Review of Clinical Psychology*, *17*(1), 495–519. 10.1146/annurev-clinpsy-081219-115923 33606946 10.1146/annurev-clinpsy-081219-115923

[CR22] Faris, R., & Ennett, S. E. (2012). Adolescent aggression: The role of peer group status motives, peer aggression, and group characteristics. *Social Networks*, *34*(4), 371–378. 10.1016/j.socnet.2010.06.003 25152562 10.1016/j.socnet.2010.06.003 PMC4138540

[CR23] Ferrari, J. R., Stevens, E. B., & Jason, L. A. (2009). The role of self-regulation in abstinence maintenance: Effects of communal living on self-regulation. *Journal of Groups in Addiction & Recovery*, *4*(1–2), 32–41. 10.1080/15560350802712371 20689650 10.1080/15560350802712371 PMC2916178

[CR24] Fromberger, P., Jordan, K., & Müller, J. L. (2018). Virtual reality applications for diagnosis, risk assessment and therapy of child abusers. *Behavioral Sciences & the Law*, *36*, 235–244. 10.1002/bsl.2332 29520819 10.1002/bsl.2332

[CR25] Gálvez-Nieto, J. L., Polanco-Levicán, K., Trizano-Hermosilla, Í., & Beltrán-Véliz, J. C. (2022). Relationships between school climate and values: The mediating role of attitudes towards authority in adolescents. *International Journal of Environmental Research and Public Health*, *19*(5), 2726. 10.3390/ijerph19052726 35270417 10.3390/ijerph19052726 PMC8910777

[CR26] Gottfredson, M. R., & Hirschi, T. (1990). *A general theory of crime*. Stanford University Press.

[CR27] Grisso, T., Barnum, R., Fletcher, K. E., Cauffman, E., & Peuschold, D. (2001). Massachusetts youth screening instrument for mental health needs of juvenile justice youths. *Journal of the American Academy of Child & Adolescent Psychiatry*, *40*(5), 541–548. 10.1097/00004583-200105000-00013 11349698 10.1097/00004583-200105000-00013

[CR28] Han, Y., Zhang, C., Huang, Y., & Zhao, L. (2024). Social comparison and aggression: The mediating role of relative deprivation and moderating role of Covert narcissism. *Current Psychology*. 10.1007/s12144-024-06013-0. Advance online publication. 10.1002/ijop.13129 38616135

[CR29] Hayes, S., Shackell, P., Mottram, P., & Lancaster, R. (2007). The prevalence of intellectual disability in a major UK prison. *British Journal of Learning Disabilities*, *35*(3), 162–167. 10.1111/j.1468-3156.2007.00461.x

[CR30] Hoogsteder, L. M., Wissink, I. B., Stams, G. J. J., van Horn, J. E., & Hendriks, J. (2014). A validation study of the brief irrational thoughts inventory. *Journal of Rational-Emotive & Cognitive-Behavior Therapy*, *32*(3), 216–232. 10.1007/s10942-014-0190-7

[CR31] Kelsey, K. R., Rogers, R., & Robinson, E. V. (2015). Self-report measures of psychopathy: What is their role in forensic assessments? *Journal of Psychopathology and Behavioral Assessment*, *37*, 380–391. 10.1007/s10862-014-9475-5

[CR32] Kip, H., Oberschmidt, K., Bierbooms, J., Dijkslag, D., Kelders, S., & Roelofsen, B. (2019). Technologie in de forensische zorg - Crossing borders. Kwaliteit Forensische Zorg. https://ris.utwente.nl/ws/files/126926528/technologie_in_de_forensische_zorg_final.pdf

[CR33] Kleeven, A. T., de Vries Robbé, M., Mulder, E. A., & Popma, A. (2022). Risk assessment in juvenile and young adult offenders: Predictive validity of the SAVRY and SAPROF-YV. *Assessment*, *29*(2), 181–197. 10.1177/1073191120959740 32964720 10.1177/1073191120959740 PMC8796163

[CR34] Klein Tuente, S., Bogaerts, S., & Veling, W. (2019). Hostile attribution bias and aggressionin adults: A systematic review. *Aggression and Violent Behavior,**46*, 66–81. 10.1016/j.avb.2019.01.009

[CR35] Koh, L. L., Day, A., Klettke, B., Daffern, M., & Chu, C. M. (2020). The predictive validity of youth violence risk assessment tools: A systematic review. *Psychology Crime & Law*, *26*(8), 776–796. 10.1080/1068316X.2020.1734200

[CR36] Krüter, I., Duindam, H. M., Asscher, J. J., & Creemers, H. E. (2023). The relationship between heart rate, heart rate variability, and aggression in detained juveniles. aggression running through your Veins?– A pilot study. *Journal of Psychophysiology*, 0. 10.1027/0269-8803/a000324

[CR37] Laninga-Wijnen, L., Harakeh, Z., Steglich, C., Dijkstra, J. K., Veenstra, R., & Vollebergh, W. (2020). The role of popular peers in the development of adolescent aggression: A longitudinal social network analysis. *Journal of Research on Adolescence*, *30*(1), 159–174. 10.1111/cdev.12650

[CR38] Lansford, J. E., Malone, P. S., Dodge, K. A., Crozier, J. C., Pettit, G. S., & Bates, J. E. (2006). A 12-year prospective study of patterns of social information processing problems and externalizing behaviors. *Journal of Abnormal Child Psychology*, *34*, 709–718. 10.1007/s10802-006-9057-4 10.1007/s10802-006-9057-4 PMC2753429 17053997

[CR39] Lochman, J. E., & Dodge, K. A. (1998). Distorted perceptions in dyadic interactions of aggressive and nonaggressive boys: Effects of prior expectations, context, and boys’ age. *Development and Psychopathology*, *10*, 495–512. 10.1017/S0954579498001710 9741679 10.1017/s0954579498001710

[CR40] Martinelli, A., Ackermann, K., Bernhard, A., Freitag, C. M., & Schwenck, C. (2018). Hostile attribution bias and aggression in children and adolescents: A systematic literature review on the influence of aggression subtype and gender. *Aggression and Violent Behavior*, *39*, 25–32. 10.1016/j.avb.2018.01.005

[CR41] Piquero, A. R., & Rocque, M. (2020). Changing self-control: Promising efforts and a way forward. *New Directions for Child and Adolescent Development*, *2020*(173), 39–47. 10.1002/cad.20368 33029851 10.1002/cad.20368

[CR42] Piquero, A. R., Jennings, W. G., Farrington, D. P., Diamond, B., & Gonzalez, J. M. R. (2016). A meta-analysis update on the effectiveness of early self-control improvement programs to improve self-control and reduce delinquency. *Journal of Experimental Criminology*, *12*, 249–264. 10.1007/s11292-016-9257-z

[CR43] Raine, A., Dodge, K., Loeber, R., Gatzke-Kopp, L., Lynam, D., Reynolds, C., Stouthamer-Loeber, M., & Liu, J. (2006). Reactive–Proactive Aggression Questionnaire (RPQ) [Database record]. *APA PsycTests*. 10.1037/t66296-000 10.1002/ab.20115 PMC2927832 20798781

[CR44] Reijntjes, A., Thomaes, S., Kamphuis, J. H., Bushman, B. J., De Castro, B. O., & Telch, M. J. (2011). Explaining the Paradoxical rejection-aggression link: The mediating effects of hostile intent attributions, anger, and decreases in state self-esteem on peer rejection-induced aggression in youth. *Personality and Social Psychology Bulletin*, *37*(7), 955–963. 10.1177/0146167211410247 21632967 10.1177/0146167211410247

[CR45] Reynolds, W. M. (1982). Development of reliable and valid short forms of the Marlowe-Crowne social desirability scale. *Journal of Clinical Psychology,**38*(1), 119–125. 10.1002/1097-4679(198201)38:1%3C119::AID-JCLP2270380118%3E3.0.CO;2-I

[CR46] Smeets, K. C., Leeijen, A. A., van der Molen, M. J., Scheepers, F. E., Buitelaar, J. K., & Rommelse, N. N. (2015). Treatment moderators of cognitive behavior therapy to reduce aggressive behavior: A meta-analysis. *European Child & Adolescent Psychiatry*, *24*, 255–264. 10.1007/s00787-014-0592-1 25138144 10.1007/s00787-014-0592-1

[CR47] Steinberg, L. (2004). Risk taking in adolescence: What changes, and why? *Annals of the New York Academy of Sciences*, *1021*(1), 51–58. 10.1196/annals.1308.005 15251873 10.1196/annals.1308.005

[CR48] Tangney, J. P., Baumeister, R. F., & Boone, A. L. (2004). High self-control predicts good adjustment, less pathology, better grades, and interpersonal success. *Journal of Personality*, *72*(2), 271–324. 10.1111/j.0022-3506.2004.00263.x 15016066 10.1111/j.0022-3506.2004.00263.x

[CR49] van der Put, C. E., Stams, G. J. J. M., Deković, M., & van der Laan, P. H. (2014). Predictive validity of The Washington state juvenile court Pre-Screen assessment in The netherlands: The development of a new scoring system. *Assessment,**21*(1), 92–107. 10.1177/1073191112436666 22333525 10.1177/1073191112436666

[CR50] Verhoef, R. E. J., Alsem, S. C., Verhulp, E. E., & De Castro, B. O. (2019). Hostile intent attribution and aggressive behavior in children revisited: A meta-analysis. *Child Development*, *90*(5), 525–547. 10.1111/cdev.13255 10.1111/cdev.13255 PMC6851691 31165477

[CR51] Verhoef, R. E. J., Van Dijk, A., Verhulp, E. E., & De Castro, B. O. (2021). Interactive virtual reality assessment of aggressive social information processing in boys with behaviour problems: A pilot study. *Clinical Psychology & Psychotherapy*, *28*(3), 489–499. 10.1002/cpp.2620 34048619 10.1002/cpp.2620 PMC8361679

[CR52] Verhoef, R. E. J., Verhulp, E. E., Van Dijk, A., & De Castro, B. O. (2022). Interactive virtual reality versus vignette-based assessment of children’s aggressive social information processing. *Research on Child and Adolescent Psychopathology*, *50*, 621–636. 10.1007/s10802-021-00879-w 34648102 10.1007/s10802-021-00879-w PMC9054903

